# Why and when suffering increases the perceived likelihood of fortuitous rewards

**DOI:** 10.1111/bjso.12406

**Published:** 2020-07-11

**Authors:** How Hwee Ong, Rob M. A. Nelissen, Ilja van Beest

**Affiliations:** ^1^ Department of Social Psychology Tilburg University The Netherlands

**Keywords:** fairness, justice, magical thinking, suffering

## Abstract

Cultural practices and anecdotal accounts suggest that people expect suffering to lead to fortuitous rewards. To shed light on this illusory ‘suffering–reward’ association, we tested why and when this effect manifests. Across three vignette studies in which we manipulated the degree of suffering experienced by the protagonist, we tested a ‘just‐world maintenance’ explanation (suffering deserves to be compensated) and a ‘virtuous suffering’ explanation (suffering indicates virtues, which will be rewarded). Our findings revealed that the illusory ‘suffering–reward’ association (1) could serve as a way for people to cope with just‐world threats posed by the suffering of innocent victims, and (2) manifested when the suffering was not caused by the victim's own behaviour and not readily attributable to bad luck. Taken together, these findings not only provide evidence for the existence of the illusory ‘suffering–reward’ association but also elucidate its psychological underpinnings.

Annually, in the early morning of May Day, students from the University of St Andrews plunge into the freezing cold North Sea, supposedly because doing so will help them obtain better grades in the upcoming examinations (University of St Andrews, 2019). A similar notion that suffering will lead to subsequent rewards also seems to be reflected in rituals that involve self‐mortification. For example, some Catholics in Philippines (Matsuzawa, 2015) and Taoists in Thailand (Mariani, 2014) self‐inflict physical suffering in hope that doing so will bring about positive outcomes such as good health and prosperity.

The notion that suffering will result in a greater likelihood of attaining future rewards is sometimes reasonable and well‐warranted. For example, suffering victims often do receive tangible help in the form of social welfare benefits and donations. Suffering may also confer intangible psychological benefits such as enhanced self‐efficacy, competence (Bastian, Jetten, Hornsey, & Leknes, 2014), and perceived meaning in life (Anderson, Kay, & Fitzsimons, 2010). However, in our current research, we focus on ‘suffering–reward’ associations in situations that are *illusory* (i.e., incompatible with the prevailing scientific understanding of cause and effect in the natural world). We put forth and test two potential psychological explanations for this association.

## ‘Just‐world maintenance’ explanation

One potential explanation (which we referred to as the ‘just‐world maintenance’ explanation) is that the illusory ‘suffering–reward’ association may serve as an effective way for people to maintain their belief in a just‐world. People have a need to believe that they are living in a world where people get what they deserve (Lerner, 1980). However, this belief could be threatened by the suffering of victims. Hence, to cope with just‐world threats, people may engage in a variety of coping strategies. One such coping mechanism is immanent justice reasoning, which involves the attribution of the victims' suffering to prior moral misconduct. For example, people may causally attribute natural disasters such as hurricanes and tsunami to prior moral misdeeds of the victims. Attributions that are characteristic of such immanent justice reasoning are generally incompatible with prevailing scientific understanding and therefore also constitute illusory associations (Callan, Sutton, Harvey, & Dawtry, 2014). However, whereas immanent justice reasoning is backward‐looking, we propose that another coping strategy may be to believe that the suffering of the innocent victim will be compensated by illusory *future* rewards.

As such, our reasoning also shares much similarity with the concept of ultimate justice reasoning, the view that injustices observed in the present are merely temporary setbacks that will be compensated in the future (Maes, 1998). However, existing research on ultimate justice reasoning has not provided clear evidence that such forward‐looking compensation can be *illusory*. For example, past research has shown that people engage in ultimate just reasoning based on their responses on the 4‐item Belief in Ultimate Justice scale (e.g., ‘In the long run, the injustice imposed by illnesses receive appropriate reparation’) developed by Maes (1998). However, the items in the scale were ambiguous with regard to the *source* of ultimate justice. That is, terms such as ‘reparation’ may be interpreted as solely stemming from sources that are well grounded in reality. Indeed, suffering victims often do receive tangible donations. For example, victims of the large‐scale Australian wildfires which started in September 2019 received an outpouring of donations in the forms of money, food, and clothes (Brown, 2020). Thus, endorsing items on the scale would not serve as evidence of an *illusory* ‘suffering–reward’ association. Reparations or compensation for suffering may also take the forms of intangible psychological rewards. Other research investigating ultimate justice reasoning has shown that people expect suffering to lead to non‐tangible rewards such as meaning, fulfilment, and purpose in life (Anderson *et al*., 2010; Harvey & Callan, 2014a, 2014b; Murayama & Miura, 2016). However, these forms of psychological rewards might also be well grounded in reality. For example, a study on residents from a town hit by tornado found that those who experienced greater exposure to the tornado and suffered greater post‐trauma stress exhibited greater post‐traumatic growth 2.5 years after the event (First, First, Stevens, Mieseler, & Houston, 2018).

Our present research would therefore go beyond existing research by addressing the open question as to whether people cope with just‐world threats by expecting that the victims will be subsequently compensated by *illusory* rewards. Specifically, we focused on rewards that take the form of fortuitous outcomes that are based on random chance (e.g., winning a lottery). In addition, we also tested another potential association as to why people may hold illusory suffering–reward associations, one that is not rooted in coping with just‐world threats.

## ‘Virtuous suffering’ explanation

Another potential explanation (which we referred to as the ‘virtuous suffering’ explanation) for the proposed illusory ‘suffering–reward’ association suggests that experiencing suffering may improve perceived moral character, which is in turn expected to bring about rewards. This explanation is derived by integrating two bodies of research literature. First, Bastian *et al*. (2014) proposed that suffering may be indicative of virtues, including those that are often associated with morality (e.g., heroism, bravery, humility). In line with this idea, several studies found that self‐punishment improves moral perceptions of oneself and by third‐party observers (Inbar, Pizarro, Gilovich, & Ariely, 2013; Nelissen, 2011; Nelissen & Zeelenberg, 2009; Zhu *et al*., 2017). Second, the enhanced moral perception may in turn be expected to bring about fortuitous rewards through an expectation that the universe will reward moral individuals (Converse, Risen, & Carter, 2012; Kulow & Kramer, 2016; Valenzuela, Bonezzi, & Szabó‐Douat, 2018; White, Norenzayan, & Schaller, 2018). As such, by proposing the ‘virtuous suffering’ explanation, we also introduce a novel explanation for the illusory ‘suffering–reward’ association that connects two largely disparate bodies of literature.

## The current research

We conducted three experiments to investigate the existence and the psychological underpinnings of the illusory ‘suffering–reward’ association. In Experiment 1, we explored if the ‘virtuous suffering’ and ‘just‐world maintenance’ mechanisms would account for the illusory ‘suffering–reward’ association. In Experiment 2, we replicated the first experiment and examined an opposing mechanism involving ‘bad luck attribution’. In Experiment 3, we examined how the inferences from suffering vary across different sources of suffering. In all, we found no support for the ‘virtuous suffering’ mechanism but obtained evidence for the ‘just‐world maintenance’ and ‘bad luck attribution’ mechanisms. The absence of a zero‐order effect of suffering on reward likelihood in experiments 1 and 2 could reflect how the two identified mechanisms working in opposing directions. Further evidence of this was provided in Experiment 3, where we attenuated the ‘bad luck attribution’ mechanism and found a zero‐order effect where suffering increased perceived reward likelihood. Together, these findings suggest that the illusory ‘suffering–reward’ association is dependent upon the interplay of a ‘bad luck attribution’ and a ‘just‐world maintenance’ mechanisms, both of which are contingent on the causes of suffering.

The three experiments in this article are presented in the order in which they were conducted. They received ethical approval from Tilburg School of Social and Behavioral Sciences's Ethics Review Board. The power analyses and results of additional analyses are available in the Supporting Information. Preregistrations, study materials, data files, and analysis scripts are available on the Open Science Framework.^1^

## EXPERIMENT 1

Experiment 1 served as an initial test of the illusory ‘suffering–reward’ association. We first manipulated the degree of suffering experienced by the protagonist in a vignette and then measured the perceived likelihood that the protagonist would receive a fortuitous reward. We further explored mediating effects of: (1) perceived *moral character* of the victim, and (2) perceived *deservingness* to receive the reward. According to the ‘virtuous suffering’ explanation, we should observe an indirect effect through perceived moral character. If the illusory ‘suffering–reward’ association serves to maintain the belief in a just‐world by balancing out the suffering with future rewards, we should observe an indirect effect through perceived deservingness.

While Lerner (1980) focused primarily on deservingness as a principle of justice (i.e., people get what they *deserve*), others emphasized another principle such as the need principle (Montada, 1998). Therefore, we also explored perceived need as another potential mediator that might also reflect the ‘just‐world maintenance’ explanation (i.e., a just‐world is one where people get what they need).

## Method

### Participants

A total of 420 Amazon Mechanical Turk workers from United States participated in the experiment. Fifty‐one participants (12%) were excluded for failing the comprehension check (*a priori* defined as answering less than three out of four comprehension check questions correctly), leaving a valid sample size of 369 (*M*_age_ = 36.09, *SD*_age_ = 11.38; 60% males, 39% females, 1% other or prefer not to say).

### Procedures and materials

Participants were first presented with a vignette introducing the protagonist's situation (see Table 1). Depending on their assigned conditions, participants read that the protagonist (Diego, a person from Venezuela) is experiencing either a great deal of suffering (high suffering condition) or relatively little suffering (low suffering condition). Next, participants read that the protagonist is eligible for the ‘green card lottery’ and had applied for it in hope of a better life. The lottery (formally known as the ‘diversity immigrant visa’) is conducted annually to diversify the immigrant population in United States by *randomly* selecting approximately 55,000 winners to receive permanent residency status.^2^ Participants in our experiment were asked to estimate the likelihood that the protagonist will win the lottery (from 0 to 100%). Next, as a manipulation check, participants rated the amount of suffering the protagonist is experiencing on a 7‐point scale (1 = *not at all*; 7 = *very much*).

**Table 1:** Vignettes used in Experiment 1

High suffering condition	Low suffering condition
Diego, a 24‐year‐old young adult living in a small town in Venezuela, is one of the many individuals trying to leave Venezuela. Despite having a high school education, Diego is unable to find stable employment. He is homeless and often has to go hungry due to the lack of money to buy food. Living on the streets, he has been the victim of several violent assaults. In hope of a better life, Diego has applied for United States’ ‘green card lottery’	Diego, a 24‐year‐old young adult living in a small town in Venezuela, is one of the many individuals trying to leave Venezuela. He owns and runs a grocery store. Business at the store is relatively good, and his earnings allow him to live in a modern house and lead a fairly comfortable lifestyle. Nonetheless, in hope of a better life, Diego has applied for United States’ ‘green card lottery’
The green card lottery, formally known as the diversity visa lottery, is intended to increase diversity in immigration. Eligible applicants are randomly selected in the lottery to receive permanent residence cards (green cards) that allow them to live and work in the United States. Approximately 55,000 green cards are awarded every year. Diego is eligible and had applied for the green card lottery.	

Participants then responded to three items measuring potential mediators on 7‐point scales. Specifically, to examine the ‘just‐world maintenance’ explanation, they first rated perceived need (‘How much do you think Diego needs to win the green card lottery?’; 1 = *not at all*, 7 = *very much*) and then deservingness (‘How much do you think Diego deserves to win the green card lottery?’; 1 = *not at all*, 7 = *very much*). To examine the ‘virtuous suffering’ explanation, participants rated perceived moral character (‘How moral do you think Diego is?’; 1 = *not moral at all*, 7 = *very moral*). As a comprehension check, participants then answered four factual questions about the protagonist's country of origin, occupation, type of housing, and financial situation. For exploratory purposes, we then administered the 7‐item Global Belief in a Just‐World scale^3^ (GBJW; Lipkus, 1991). Finally, participants provided information on age, gender, and political ideology.

## Results

### Manipulation check

We were successful in manipulating the protagonist's level of suffering. Participants in the high suffering condition rated the protagonist as experiencing greater levels of suffering (*M* = 5.83, *SD* = 1.19) than those in the low suffering condition (*M* = 2.90, *SD* = 1.59), *t*(327) = 19.89, *p* < .001, *d* = 2.09, CI_95%_ [1.84, 2.35].

### Zero‐order effects of suffering on reward likelihood

Suffering did not increase the perceived likelihood of fortuitous rewards. An independent sample *t*‐test indicated that participants in the high suffering condition (*M* = 14.26%; *SD* = 19.97%) did not expect the protagonist to be more likely to win the green card lottery compared to participants in the low suffering condition (*M* = 18.66%, *SD* = 22.8%). Instead, there was a marginally significant effect in the opposite direction, *t*(353) = 1.97, *p* = .050, *d* = 0.21, CI_95%_ [0.00, 0.41].

### Exploratory mediation analyses

**Figure 1:**
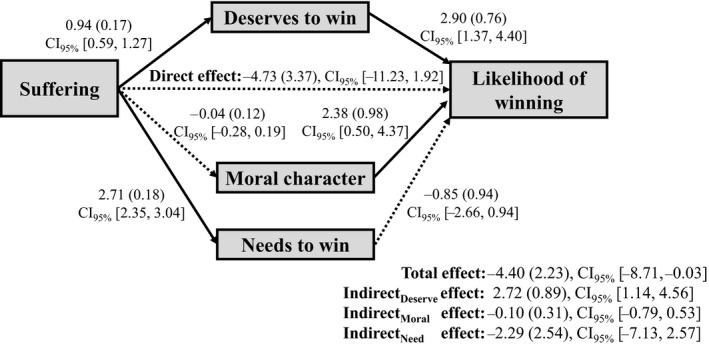
Results of mediation analysis with all three mediators concurrently. Unstandardized coefficients shown with standard errors in parentheses. Solid line denotes significant path, while dashed line denotes non‐significant path.

To probe for potential mediation effects, we tested a mediation model with suffering as the independent variable; perceived moral character, deservingness, and need as concurrent mediators; and perceived likelihood of winning the lottery as the outcome variable. Mediation analyses in this experiment were conducted using R package *lavaan* version 0.6‐3 (Rosseel, 2012) – standard errors were estimated with 5,000 bootstrap draws. As seen in Figure 1, the indirect effect of suffering on the likelihood of winning through deservingness was significant in the positive direction but the indirect effects through moral character and need were not. This pattern of findings held up in additional mediation analyses where each of the three mediators was included in separate models (see Supporting Information).

## Discussion

The result indicated that the level of suffering experienced by the protagonist did not increase the perceived likelihood that the protagonist would receive a fortuitous reward (i.e., winning the green card lottery). Nonetheless, even when zero‐order effects are absent, there may still be significant indirect effect(s). As an illustration, a researcher interested in the effects of intelligence on task performance might observe no zero‐order effect (McFatter, 1979; Rucker, Preacher, Tormala, & Petty, 2011). However, there might still be two opposing indirect effects. First, intelligence might be associated with greater ability, which in turn led to better performance (i.e., intelligence → greater ability → better performance). Second, intelligence might also have resulted in greater boredom, which in turn led to poorer performance (i.e., intelligence → greater boredom → worse performance). As such, the mere absence of a significant zero‐order effect should not prevent the further exploration of the psychological processes underlying a presumed phenomenon (Rucker *et al*., 2011).

Indeed, in our experiment, we found a significant indirect effect through deservingness that provided initial support for the ‘just‐world maintenance’ explanation. The presence of a significant indirect effect without a significant zero‐order effect may indicate the presence of an overlooked opposing mechanism that was not included in the mediation model (Zhao, Lynch, & Chen, 2010). However, our findings did not support the ‘virtuous suffering’ explanation. There was neither a significant indirect effect via perceived moral character, nor did suffering improve perceived moral character.

The results we obtained might have been influenced by some idiosyncratic aspects of the vignette. As the participants were from United States, whether or not the protagonist wins the green card lottery could be construed as being personally relevant to the participants. That is, if the protagonist wins the green card lottery, he will be immigrating to the participants' country of residence. As homeless individuals might be perceived less favourably and elicit more negative emotions such as contempt (Fiske, Cuddy, Glick, & Xu, 2002), participants in the high suffering condition who read that the protagonist is homeless might prefer for the protagonist to *not* win the lottery. This preference could in turn lower the perceived likelihood that the protagonist would win the lottery due to wishful thinking (also known as the desirability bias; Krizan & Windschitl, 2009). This process could have contributed to the negative direct effect in our mediation models where suffering reduced the perceived likelihood of winning the lottery. In our subsequent experiments (experiments 2 and 3), this idiosyncrasy is circumvented as the vignettes were written such that the outcomes of the protagonists were of no obvious personal relevance to the participants.

## EXPERIMENT 2

Experiment 2 built upon the initial findings of Experiment 1 in several ways. First, we examined an additional mechanism revolving around ‘bad luck attribution’ in which suffering may also *decrease* the perceived likelihood of fortuitous rewards. To clarify, certain conceptualizations of luck (i.e., ‘stable luck’) refer to luck as an internal attribute of a person that it is relatively stable (Maltby, Day, Gill, Colley, & Wood, 2008). According to this view, an individual who is currently unlucky will continue to be unlucky in the future. It might be the case that participants in Experiment 1 had perceived the protagonist who is experiencing greater suffering to be unluckier and that this unluckiness had translated to a lower perceived likelihood that he would subsequently win the green card lottery. Second, we aimed to replicate our finding which supported the ‘just‐world maintenance’ explanation. Third, in order to examine the robustness of the findings in Experiment 1 across different measurement methods, we employed (1) an additional measure of reward likelihood, and (2) a more comprehensive measure of moral character.

## Method

### Participants

A total of 539 Amazon Mechanical Turk workers from United States participated in the experiment. Forty‐seven (9%) participants were excluded for failing the comprehension check (*a priori* defined as answering less than three out of four comprehension check questions correctly), leaving a valid sample size of 492 (*M*_age_ = 36.43, *SD*_age_ = 11.13; 53% males, 46% females, 1% other or prefer not to say).

### Procedures and materials

Participants were presented with a vignette shown in Table 2. Depending on their assigned conditions, participants either read that the protagonist, a person with cleft lip named Diego, is currently experiencing either a great deal of suffering (high suffering condition) or not (low suffering condition). Next, participants read that the protagonist has been entered into a random draw to potentially receive free medical treatment for his cleft lip. Participants were then asked to estimate the likelihood that the protagonist will be selected in the random draw on (1) a percentage scale (from 0 to 100%) and (2) 7‐point scale (1 = *very low chance to be selected*, 7 = *very high chance to be selected*).

**Table 2:** Vignettes used in Experiment 2

High suffering condition	Low suffering condition
Diego is a 14‐year‐old teen in Venezuela with cleft lip. Cleft lip is a form of birth defect where a baby's lip does not form properly during pregnancy. Due to the lack of accessible health care in his country, Diego, like many others with the same condition, was not able to receive treatment for his condition.	
Because of this medical condition, Diego has been the target of vicious bullying. He has no friends and is beaten by the bullies from time to time. He often feels anxious, insecure, and lonely.	Despite this medical condition, Diego has a relatively healthy social environment. He has several good friends whom he frequently hangs out with. He is usually cheerful and contended with his life.
Recently, it was announced that a volunteer medical team from abroad will be arriving in a nearby city to offer free corrective surgery for patients with Diego's condition. Diego is one of several hundred applicants who signed up to receive the free treatment. However, due to the medical team's limited time and resources, they are only able to offer treatment to several dozen patients. The team therefore decided to conduct random draws to decide who gets to receive the surgery.	

As a manipulation check, participants rated the degree of suffering the protagonist is experiencing on a 7‐point scale. Participants then responded to three items, each measuring a potential mediator on a 7‐point scale (1 = *not at all*, 7 = *very much*): (1) need (‘How much do you think Diego needs the surgery?’ (2) deservingness (‘How much do you think Diego deserves to be selected for the surgery?’), and (3) unluckiness (‘How unlucky do you think Diego is?’). Next, we measured perceived moral character using a 6‐item measure used in prior research (Goodwin, 2015; Landy, Piazza, & Goodwin, 2016). Specifically, participants were asked to rate the protagonist on six traits (moral, principled, honest, trustworthy, fair, and responsible) on a 7‐point scale (1 = *not at all*, 7 = *very much*). This measure exhibited good internal consistency reliability (*α* = .97) for our sample.

As a comprehension check, participants answered four factual questions about the protagonist's country of origin, relationship with peers, usual mood, and the way patients will be selected for the surgery. Participants then filled out the same GBJW scale (Lipkus, 1991) used in Experiment 1. Finally, participants provided demographic information (i.e., age and gender).

## Results

### Manipulation check

We were successful in manipulating the protagonist's level of suffering. Participants in the high suffering condition rated the protagonist as experiencing greater levels of suffering (*M* = 5.84, *SD* = 1.07) than those in the low suffering condition (*M* = 3.46, *SD* = 1.45), *t*(449) = 20.70, *p* < .001, *d* = 1.87, CI_95%_ [1.66, 2.08].

### Zero‐order effects of suffering on reward likelihood

As in Experiment 1, suffering did not increase the perceived likelihood of receiving fortuitous rewards. Independent sample *t*‐tests indicated that the participants in the high and low suffering conditions did not significantly differ on both the percentage and 7‐point measures of the perceived likelihood of fortuitous rewards (see Table 3).

**Table 3:** Comparison of the perceived likelihood of reward across conditions

Dependent variable	Condition		*t*‐test statistics			
	High suffering	Low suffering	*t*	*p*	*d*	CI_95%_
Likelihood (percentage)	*M* = 27.97 % *SD* = 21.08 %	*M* = 26.42 % *SD* = 20.29 %	*t*(490) = 0.83	.408	0.07	[−0.10, 0.25]
Likelihood (7‐point)	*M* = 3.18 *SD* = 1.31	*M* = 3.18 *SD* = 1.27	*t*(490) = 0.02	.982	0.00	[−0.18, 0.18]

### Mediation analyses

**Figure 2:**
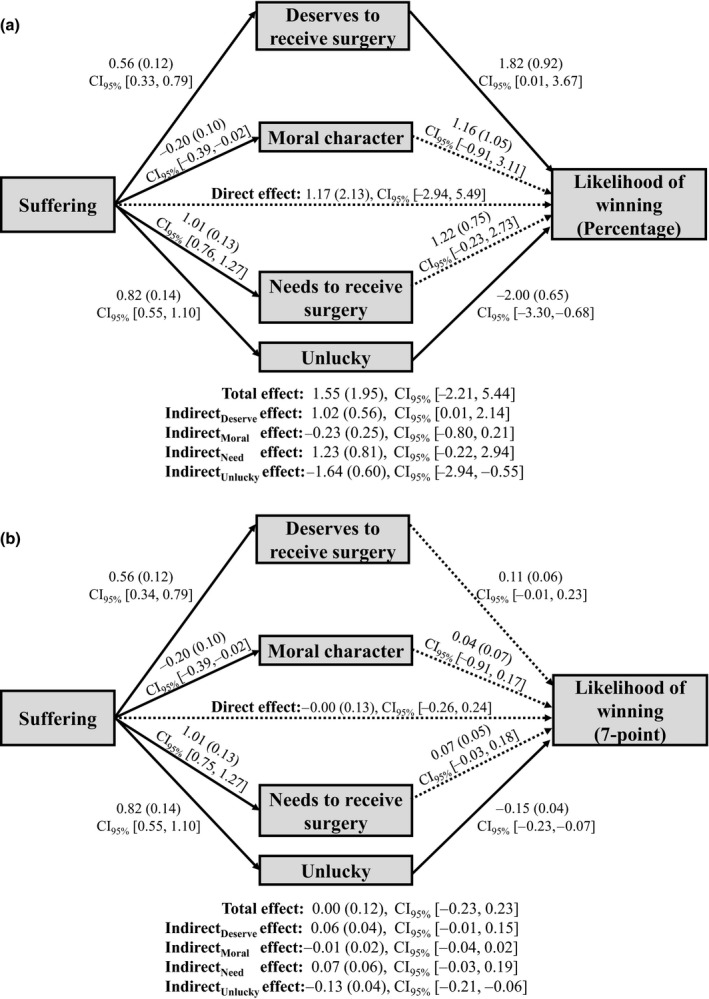
Results of mediation analyses with all four mediators concurrently with (a) percentage measure of reward likelihood and (b) 7‐point measure of reward likelihood. Unstandardized coefficients shown with standard errors in parentheses. Solid line denotes significant path, while dashed line denotes non‐significant path.

To probe potential mediation effects, we first tested a mediation model, with suffering as the independent variable; deservingness, need, moral character, and unluckiness as concurrent mediators; and perceived reward likelihood as the outcome variable. Mediation analyses in this experiment were conducted using R package *lavaan* version 0.6‐3 (Rosseel, 2012) – standard errors were estimated with 5,000 bootstrap draws. Results are shown in Figure 2. We found no significant indirect effect of suffering on likelihood through need and moral character. We did, however, find a significant indirect effect through unluckiness. That is, the protagonist who is experiencing higher level of suffering was perceived to be unluckier, which was in turn associated with lower perceived reward likelihood.

**Figure 3:**
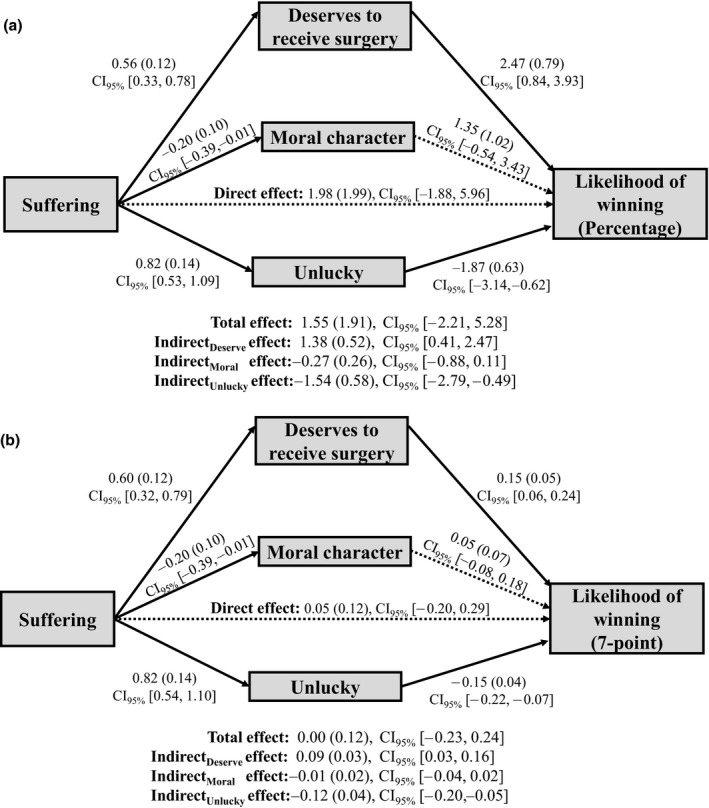
Results of mediation analyses with deservingness, moral character, and unluckiness as concurrent mediators. Reward likelihood is measured on a (a) percentage measure and (b) 7‐point measure. Unstandardized coefficients shown with standard errors in parentheses. Solid line denotes significant path, while dashed line denotes non‐significant path.

As in Experiment 1, we also found a positive indirect effect through deservingness that was significant for the percentage measure of reward likelihood but only marginally significant for the 7‐point measure. We speculated that this marginally significant finding might be due to a conceptual overlap between need and deservingness. As noted by Preacher and Hayes (2008), correlated mediators can ‘compromise the significance of particular indirect effects’ (p. 882). Thus, the relatively high correlation between need and deservingness in this experiment (*r* = .573, *p* < .001) could have attenuated the indirect effect through deservingness. Further, prior research had also identified need perceptions as an important factor that underlies deservingness judgement (Lamm & Schwinger, 1980; Skitka & Tetlock, 1992; Taormina & Messick, 1983). Therefore, we reasoned that it may not be statistically and theoretically sound to concurrently include deservingness and need in the same mediation model. Indeed, excluding need perception as a mediator resulted in significant indirect effects through deservingness and unluckiness for both measures of rewards likelihood (see Figure 3).^4^

## Discussion

The findings in this experiment are consistent with those of Experiment 1. While our suffering manipulation had no zero‐order effect on reward likelihood, we found the same mediating effect of deservingness where individuals who experienced greater levels of suffering were perceived to be more deserving of future rewards, which was in turn associated with a higher likelihood of fortuitous rewards. As before, our findings did not provide support for the ‘virtuous suffering’ explanation. In support of the ‘bad luck attribution’ explanation, we found a mediating effect through unluckiness. That is, people perceived individuals who experienced greater levels of suffering to be unluckier and this unluckiness was in turn associated with a lower likelihood of fortuitous rewards.

## EXPERIMENT 3

In Experiment 2, we found evidence for two competing psychological mechanisms that might underlie the illusory ‘suffering–reward’ association, albeit in opposing directions, namely (1) a ‘just‐world maintenance’ mechanism that increases the perceived reward likelihood, and (2) a ‘bad luck attribution’ mechanism that decreases the perceived reward likelihood. In Experiment 3, we aimed to corroborate and build upon these findings by examining how the interplay of these two competing mechanisms varies with different *causes* of suffering.

We differentiated between three types of causes: (1) other individuals, (2) the self, and (3) stochastic processes. We reasoned that when there is an obvious cause for the suffering, such as it being clearly attributable to other individuals or the self, people are less likely to attribute the suffering to bad luck, thereby attenuating the ‘bad luck attribution’ mechanism. In addition, we expected that the ‘just‐world maintenance’ mechanism would be attenuated when suffering is perceived to be deserved (e.g., when it is caused by oneself).

The above reasonings led us to make several predictions regarding the indirect effects through deservingness and unluckiness for each of the three causes of suffering (see also Table 4 for an overview). First, when suffering is caused by other individuals and perceived as undeserved, we expected the ‘bad luck attribution’ mechanism, but not the ‘just‐world maintenance’ mechanism, to be attenuated. Second, when suffering is caused by oneself, we also expected the ‘bad luck attribution’ mechanism to be attenuated due to the presence of a clear cause. Because the suffering resulted from one's own decisions and behaviour, the victim is likely to be perceived as being responsible for and deserving of the suffering. This is expected to pose minimal threats to just‐world beliefs, thereby attenuating the ‘just‐world maintenance’ mechanism. Third, when undeserved suffering is the result of stochastic (i.e., random) processes, we expected that neither the ‘bad luck attribution’ nor the ‘just‐world maintenance’ mechanisms would be attenuated.

**Table 4:** Overview of the predictions for various causes of suffering

Cause of suffering	Strength of mechanism		Zero‐order effect of suffering on reward likelihood
	Just‐world maintenance (positive effect)	Bad luck attribution (negative effect)	
Other individuals	Stronger	Weaker	Positive
Self	Weaker	Weaker	Weak or absent
Stochastic processes	Stronger	Stronger	Weak or absent

As the two mechanisms were expected to act in opposite directions, the zero‐order order effect of suffering on reward likelihood should reflect the *aggregation* of both indirect effects (see Table 4). Thus, we predicted that a positive zero‐order effect would emerge only when suffering is caused by others. While different patterns of indirect effects were expected for suffering caused by the self and stochastic processes, we predicted that both forms of suffering would exert weak or no zero‐order effect.

## Method

Using a between‐subject design, we manipulated the cause of suffering experienced by the protagonist in a vignette. Participants read that the protagonist either experienced suffering caused by another individual (other condition), caused by the self (self‐condition), resulting from stochastic processes (stochastic condition), or did not experience suffering (control condition). Participants then estimated the likelihood that the protagonist will subsequently experience a positive fortuitous outcome.

### Participants

A total of 1,619 Amazon Mechanical Turk workers from United States participated in the experiment. Ninety‐five participants (5.8%) were excluded for failing the comprehension check (*a priori* defined as answering less than two out of three comprehension check questions correctly), leaving a valid sample size of 1,524 (*M*_age_ = 36.08, *SD*_age_ = 11.71; 49.9% males, 49.5% females, 0.6% other or prefer not to say).

### Procedures

Participants were presented with the vignette (see Table 5) selected from a pre‐test (see Supporting Information for information on the pre‐test). The protagonist in this vignette is a university student majoring in French. Depending on the participants' assigned conditions, they either read that the protagonist is experiencing suffering (i.e., limb amputation) that was caused by another individual (other condition), caused by his own decision (self‐condition), the result of stochastic processes (stochastic condition), or is not experiencing suffering (control condition). Next, participants read that the protagonist had applied for a study abroad programme in France but that it was oversubscribed, and that the vacancies will be allocated via a random draw. Participants then estimated the likelihood that the protagonist will be selected for the study abroad programme on the 7‐point measure (1 = *very low chance*, 7 = *very high chance*).

**Table 5:** Vignette (‘amputation’) used in the four conditions of Experiment 3

Conditions			
Other	Self	Stochastic	Control
Alan is currently a 21‐year‐old junior at university. He is majoring in French and has great interest in French literature and culture.			
Alan is also a motorcycle enthusiast who frequently brought his fancy motorcycle out for a spin around his college town whenever he felt stressed out from school work. However, that changed sometime last year when he crashed his motorcycle into a tree. He got seriously injured and doctors had to amputate his left leg below the knee in order to save his life			Alan is also a motorcycle enthusiast, frequently bringing his fancy motorcycle out for a spin around his college town whenever he feels stressed out from school work
Police investigation revealed that the crash was the result of a love rival sabotaging the brakes of Alan's motorcycle	Police investigation revealed that the crash was the result of Alan riding under the influence of alcohol	Police investigation revealed that the crash was due to a falling branch that hit Alan, causing him to momentarily lose control of his motorcycle	**–**
The amputation took a heavy emotional toll on Alan. He has been experiencing great grief and is constantly worried about how other people will view him. Nonetheless, he resumed his education several months later			–
Recently, Alan received a notice from the university informing students about the possibility to go for a 6‐month study abroad programme in France. Alan is very excited about this as he has dreamt of visiting France since high school. He believes that the trip will also help him immerse himself in the culture he has been learning so much about in class. He applied for the programme and eagerly awaits the outcome. It turns out that there are several dozen eligible applicants but only eight vacancies. Therefore, the university will conduct a random draw to allocate the vacancies			
What do you think Alan's chance of being selected for the study abroad programme is?			

Next, participants rated the protagonist's deservingness to be selected for study abroad programme (1 = *not at all*, 7 = *very much*) and how unlucky he is (1 = *not at all*, 7 = *extremely*). The perceived moral character of the protagonist was measured using the same 6‐item measure used in Experiment 2. This measure exhibited good internal consistency reliability (*α* = .95) for our sample. As a manipulation check, participants rated the degree of suffering the protagonist is experiencing (1 = *not at all*, 7 = *very much*). As a comprehension check, participants answered three factual questions about the vignette. Finally, participants provided demographic information (i.e., age and gender).

## Results and discussion

Descriptive statistics of the key variables are presented in Table 6.

**Table 6:** Means and standard deviations of key variables in Experiment 3

Variable	Condition			
	Control	Other	Self	Stochastic
Reward likelihood	3.81 (1.29)	4.02 (1.40)	3.73 (1.23)	4.11 (1.35)
Deserve	5.31 (1.16)	5.51 (1.19)	4.70 (1.34)	5.33 (1.26)
Moral	5.10 (0.98)	5.22 (1.08)	4.04 (1.15)	5.12 (0.99)
Unlucky	3.29 (1.13)	4.47 (1.48)	3.59 (1.47)	4.60 (1.54)
Suffering	2.68 (1.39)	5.86 (1.00)	5.46 (1.19)	5.64 (1.17)

Note

Means are presented with standard deviations in parentheses. Variables were measured on a 1‐ to 7‐point scale.

### Manipulation check

We were successful in manipulating the protagonist's level of suffering. The perceived suffering experienced by the protagonist was significantly higher in the three suffering conditions (*M*s ranging from 5.46 to 5.86) as compared to the control condition (*M* = 2.68), all *p*s < .001.

### Zero‐order effects of suffering on reward likelihood

Participants in the four conditions differed in the perceived likelihood of fortuitous rewards. An one‐way ANOVA indicated that reward likelihood differed across the four conditions, *F*(3, 1,520) = 7.33, *p* < .001, η^2^ = .014, CI_95%_ [0.004, 0.028]. Results of the corresponding pairwise *t*‐tests are shown in Table 7. As predicted, we found that the perceived reward likelihood in the other condition was higher than that in the control condition. Contrary to our prediction, the perceived reward likelihood in the stochastic condition was also higher than that of the control condition. Perceived reward likelihood did not significantly differ between the control and self‐conditions.

**Table 7:** Results of pairwise *t*‐tests comparing the perceived reward likelihood in the three suffering conditions with that in the control condition

Comparison	*t*‐test statistics				
	*t*	*df*	*p*	*d*	CI_95%_
Other versus control	2.07	688	.039	0.16	[0.01, 0.30]
Stochastic versus control	3.24	782	.001	0.23	[0.09, 0.37]
Self versus control	0.89	765	.373	−0.06	[−0.21, 0.08]

Note

These statistics were based on non‐pooled variance, but using pooled variance led to the same pattern of results.

### Mediation analyses

We tested a mediation model with (1) experimental condition as a multinomial independent variable (with control condition as the reference group), (2) deservingness, unluckiness, and moral character as concurrent mediating variables, and (3) perceived reward likelihood as the outcome variable. The mediation model was tested with jamovi's jAMM module^5^ (The jamovi project, 2019) using the bootstrap (percentile) method with 5,000 draws to estimate the standard errors. Results are shown in Table 8. As in experiments 1 and 2, we did not find support for the ‘virtuous suffering’ explanation. We now turn to examine support for our predictions relating to the mechanisms involving ‘just‐world maintenance’ and ‘bad luck attribution’.

**Table 8:** Results of mediation analysis in Experiment 3

Suffering condition (vs. control)	Indirect effect			Direct effect	Zero‐order effect
	via Deserve	via Unlucky	via Moral		
Other	0.059* (0.026) CI_95%_ [0.010, 0.112]	−0.048 (0.031) CI_95%_ [−0.109, 0.012]	0.006 (0.006) CI_95%_ [−0.004, 0.021]	0.195 (0.104) CI_95%_ [−0.010, 0.398]	0.213* (0.099) CI_95%_ [0.019, 0.407]
Self	−0.179* (0.033) CI_95%_ [−0.247, −0.117]	−0.012 (0.009) CI_95%_ [−0.032, 0.003]	−0.050 (0.038) CI_95%_ [−0.124, 0.025]	0.163 (0.095) CI_95%_ [−0.024, 0.349]	−0.077 (0.095) CI_95%_ [−0.263, 0.108]
Stochastic	0.006 (0.025) CI_95%_ [−0.044, 0.055]	−0.053 (0.034) CI_95%_ [−0.120, 0.013]	0.001 (0.004) CI_95%_ [−0.007, 0.010]	0.356* (0.094) CI_95%_ [0.168, 0.537]	0.310* (0.094) CI_95%_ [0.125, 0.495]

Note

Unstandardized coefficients are presented, with standard errors in parentheses.

* CI does not overlap with 0.

#### Other‐caused suffering

We predicted that when suffering was caused by another individual, we would observe a significant positive indirect effect through deservingness and no or weak indirect effect through unluckiness. These two predictions were supported (see Table 8).

#### Self‐caused suffering

We predicted that when suffering was caused by oneself, the indirect effect through deservingness would not be significant. Contrary to our prediction, we found such an indirect effect in the *negative* direction: suffering *decreased* deservingness, which was in turn positively associated with perceived reward likelihood. As predicted, there was no significant indirect effect through unluckiness.

#### Stochastic suffering

We had predicted significant indirect effects through deservingness and unluckiness. However, contrary to our predictions, both indirect effects were not significant. We did, however, observe a positive zero‐order effect, suggesting that there could be other mechanism(s) at work.

## GENERAL DISCUSSION

We set out to find formal empirical support for an illusory ‘suffering–reward’ association and to examine the underlying mechanisms and the conditions of its occurrence. Across three experiments with different operationalizations of suffering, we tested three psychological mechanisms. The first mechanism, which we termed ‘virtuous suffering’, draws on and connects two largely disparate bodies of research. One body of research was based on the notion that experiencing suffering would lead the victim to be perceived as more moral (Bastian *et al*., 2014). The enhanced moral character might in turn result in the victim being perceived as more likely to receive fortuitous rewards through the tendency to expect good things to happen to good people (White *et al*., 2018). However, our findings did not support this explanation. Across all three experiments, perceived moral character did not mediate the effects of suffering on reward likelihood as would be expected by this explanation.

On the other hand, we found support for what we termed the ‘just‐world maintenance’ explanation. This explanation was based on prior work suggesting that people have a need to believe that the world is just and that this need would be threatened by undeserved suffering in the world (Lerner, 1980). We proposed that expecting suffering to be subsequently compensated by *fortuitous* rewards could serve as an alternative way for people to mitigate just‐world threats. Across all three experiments, we found that perceived deservingness mediated the effects of suffering on reward likelihood. That is, suffering victims were perceived as more deserving of future reward and this increased deservingness was in turn associated with a greater perceived likelihood to actually receive the reward. Because this explanation hinges upon suffering posing a just‐world threat, we reasoned that this effect ought to be absent when suffering was caused by oneself as the suffering would be perceived as deserved and not pose any just‐world threat. This reasoning was supported by the results of Experiment 3.

Our findings therefore contribute towards our understanding of how people cope with just‐world threats by going beyond commonly studied coping strategies such as victim‐blaming and victim‐derogation (Lerner, 1980). It also lends support to the notion that people can cope with just‐world threats by engaging in ultimate justice reasoning (i.e., thinking that present injustices will be compensated in the future). While previous research did not provide clear evidence that the compensation in ultimate justice reasoning can be illusory, our findings fill this void by demonstrating that people may expect injustices (e.g., undeserved suffering) to be compensated by future *illusory* rewards.

Our experiments had largely focused on situations where suffering is compensated with rewards that directly address the suffering. For example, winning the green card lottery could alleviate the suffering of a homeless individual, just as corrective surgery for cleft lip would for a patient with the medical condition. We propose that these forms of ‘within‐domain’ effects where the reward befits the suffering could be the most prototypical examples of justice being served. As such, we expect ‘within‐domain’ effects to be most pronounced. Nonetheless, our third experiment where a misfortune in the health domain (i.e., limb amputation) could be compensated by a reward in the education domain (i.e., selected for study abroad programme) appears to provide some evidence of cross‐domain effects. Our findings also established the presence of another mechanism involving ‘bad luck attribution’. According to this explanation, victims of suffering might be perceived to be unluckier and this perceived bad luck could then translate to a lower perceived likelihood of obtaining fortuitous rewards. Consistent with this explanation, we found in Experiment 2 that suffering decreased the perceived likelihood of fortuitous rewards through unluckiness. We further predicted that this mechanism would be attenuated when suffering had a clear cause (e.g., caused by the self or others). This prediction was bore out in Experiment 3, lending further support to this explanation.

Our research also provided insights on suffering that resulted from stochastic processes. In Experiment 3, we found that this form of suffering increased the perceived likelihood of fortuitous rewards. Intriguingly, while this indicated the presence of the illusory ‘suffering–reward’ association, mediation analyses indicated that this zero‐order effect was not explained by any of the three above‐mentioned mechanisms. While the underlying mechanism(s) remains an open question, we propose that a possible candidate is a mechanism similar to that which underlies the gambler's fallacy (Burns & Corpus, 2004). Akin to the tendency to fallaciously believe that a ‘head’ on a fair coin toss is more likely to be followed by a ‘tail’, people may construe stochastic life outcomes in a similar manner and expect positive fortuitous events (e.g., getting selected for the exchange programme) to be more probable after negative events (e.g., losing limb due to stochastic processes). Crucially, such a mechanism would speak to a more general phenomenon than our present focus on the illusory ‘suffering–reward’ association, reflecting how people perceive randomness and chance in everyday life.

Beyond illuminating the underlying mechanisms of the illusory ‘suffering–reward’ association, our findings also highlight the importance of looking beyond zero‐order effects. As pointed out by Rucker *et al*. (2011), focusing solely on zero‐order effects may ‘cause researchers to miss theorized relationships that are present in the data’ (p. 368). Given the complexity of the human psyche, it should come as no surprise that a multi‐faceted psychological construct such as suffering can influence judgement through more than one mechanism. Indeed, the examination of mediating effects offered a better appreciation of how the illusory ‘suffering–reward’ association reflects the interplay of two opposing psychological mechanisms.

### Limitations

Inherently, a mediation model only tests one of several possible causal models and cannot rule out reverse mediation models (Fiedler, Schott, & Meiser, 2011; Thoemmes, 2015). Nonetheless, we believe that our interpretation of the findings is strengthened by our findings in Experiment 3 where the indirect effect through deservingness varied across different causes of suffering in a theoretically expected manner (i.e., the indirect effect was absent when suffering was self‐caused). Deservingness, as a mediator, also differed across the causes of suffering (e.g., deservingness was lower when suffering was caused by oneself). This systematic variation of the mediator would allay concerns regarding confounding in mediation analysis (Rohrer, 2019).

While our results did not provide support for the ‘virtuous suffering’ mechanism, we acknowledge that we cannot rule out the possibility that this mechanism may emerge under other condition(s). For instance, suffering may improve perceived moral character when it is exceptionally severe, experienced in the pursuit of a worthy cause, or when it has been successfully overcome.

While we had focused on testing the ‘virtuous suffering’ and ‘just‐world maintenance’ explanations, a more complex explanation that is a hybrid of the two may nonetheless remain theoretically plausible. Specifically, suffering could have enhanced moral character, which would in turn increase perceived deservingness and subsequently reward likelihood (i.e., a serial mediation model: suffering → moral character → deservingness → reward likelihood). However, this explanation was not borne out by our results as suffering did *not* enhance perceived moral character in any of the three experiments.

The participants in all three experiments were Amazon's Mechanical Turk workers from United States. While the generalizability of our findings beyond Americans remains undemonstrated, we believe that the fact that we found support for our key finding regarding ‘just‐world maintenance’ in samples with relatively strong meritocratic beliefs and individualistic values that emphasizes personal responsibilities presents a strong case that it would generalize to other cultures. Despite a possible inclination to hold individuals responsible and accountable for their negative outcomes and causally attribute suffering to the victims, we nonetheless found support for the illusory ‘suffering–reward’ association, suggesting that the association could be even more prominent in cultures with less emphasis on personal responsibility.

### Implications and future directions

We often rely on our forecasts of future outcomes when making decisions. Thus, when forecasts about an individual's outcome are influenced by the degree of suffering experienced by the individual, sub‐optimal decision‐making might ensue. Our current research thus paves the way for future research to investigate potential implications of the illusory ‘suffering–reward’ association. For example, if people expect that their personal suffering would be compensated in implausible ways, they may be unrealistically optimistic in their forecasts and thus engage in maladaptive risky behaviour (e.g., financial investment, dangerous stunts). Another potential implication is its effects on helping behaviour. If people expect suffering victims to be compensated in the absence of any such indication, this ‘everything will be okay’ mentality might lead to a reduced tendency to personally render aid.

### Conclusion

Our research furthers our understanding of why and when people may hold an illusory ‘suffering–reward’ association. We found that the illusory association results from the interplay of two opposing psychological mechanisms. The first, which involves the tendency to expect suffering to be compensated in unwarranted ways, manifests when suffering is undeserved. The other, which involves the attribution of suffering to bad luck, emerges when there is no obvious cause to the suffering.

## Conflicts of interest

All authors declare no conflict of interest.

## Author contribution

How Hwee Ong (Conceptualization; Formal analysis; Methodology; Writing – original draft; Writing – review & editing); Rob M. A. Nelissen (Conceptualization; Methodology; Supervision; Writing – review & editing); Ilja van Beest (Conceptualization; Funding acquisition; Methodology; Supervision; Writing – review & editing).

## Supporting information


**Appendix S1.** Online Supplementary Materials.

## Data Availability

The data associated with this manuscript are available at the following OSF repository: https://osf.io/5t47x/?view_only=e35dd68fe30744129d21e5940c930164. This private link is currently anonymized for peer review. Following the policy of our university, we will make the data and materials available upon acceptance of our manuscript.

## Footnotes

^1^
https://osf.io/5t47x/

^2^ The vignettes in our current set of experiments included some guiding information (e.g., participants were informed that 55,000 individuals are selected of the green card lottery) in order to reduce the variability in participants' probability estimates. The information provided is insufficient for participants to calculate the exact probability of receiving the positive outcome.

^3^ Results of moderated mediation analyses with GBJW and political ideology as moderators are reported in the Supporting Information.

^4^ We had also tested additional mediation models where each of the four mediators was included in separate models. Briefly, we found evidence for indirect effects through deservingness and need in the positive directions, and through unluckiness in the negative direction (see Supporting Information for details).

^5^ jamovi's jAMM module also utilizes the R package *lavaan* to estimate mediation models. We used jamovi in Experiment 3 because it provides a graphical user interface which we believe would reduce inadvertent errors when specifying relatively complex mediation model.
