# Supplementary material for: Why and when suffering increases the perceived likelihood of fortuitous rewards
Source: Br J Soc Psychol. 2020 Jul 11;60(2):e12406. doi: 10.1111/bjso.12406 (PMC8048465; doi:10.1111/bjso.12406)
Supplement: Supplementary file 1 — Appendix S1. Online Supplementary Materials. [file BJSO-60-548-s001.docx]

**ONLINE SUPPLEMENTARY MATERIALS**

How Hwee Ong, Rob Nelissen, Ilja van Beest

Last updated: 6 July 2020

- This is the online supplementary materials for the article “*Why and When Suffering Increases the Perceived Likelihood of Fortuitous Rewards”*
- This project has an Open Science Framework (OSF) page at: <https://osf.io/5T47X/>
- The OSF page contains materials (e.g., Qualtrics survey, preregistration), deidentified data, analysis scripts, and output.

**Content**

**Experiment 1**

Method ……………………………………………………………….…. 2

Analyses and Results …………………………………………….……… 3 – 6

**Experiment 2**

Method ………………………………………………………………….. 7

Analyses and Results ……………………………………………………. 7 – 11

Brief Discussion …………………………………………………………. 12

**Experiment 3**

Method …………………………………………………………………… 13 – 14

Analyses and Results ……………………………………………………. 14

**Experiment 3 Pretest**

Method …………………………………………………………………… 15 – 16

Analyses and Results ……………………………………………………. 16 – 18

Brief Discussion ……………………………………………………….… 19

Appendix A ……………………………………………………….…...………… 20 – 23

References ……………………………………………………….…...………….. 24

**Experiment 1**

**Method**

**Sample size computation.** Sensitivity power analysis conducted using G*Power (Faul, Erdfelder, Buchner, & Lang, 2009) indicated that with a sample size of 350, we had 80% power to detect an effect size of *d* = 0.30 in a two-tailed independent sample *t*-test at an alpha level of .05. We deemed this level of power to be adequate. Our experiment contained four comprehension check questions and we *a priori* decided and preregistered that we would exclude participants who failed the comprehension check (i.e., answered less than three out of four questions correctly) from analyses. To account for exclusion, we adjusted our target sample size upwards by 20%, resulting in a target sample size of 420.

**Data collection.** Participants were Amazon Mechanical Turk (MTurk) workers recruited using TurkPrime. To participant in the experiment, participants needed an approval rate of at least 90% and had to be from United States. At the time this experiment was conducted, there were concerns about low quality data from MTurk workers (e.g., from bots). We attempted to mitigate this concern by taking several measures: (i) incorporating Google’s reCAPTCHA which participants had to pass at the start of the experiment, and (ii) enabling TurkPrime’s “block duplicate IP addresses”, “block duplicate geolocation”, and “block suspicious geocode locations” features.

**Analyses and Results**

The correlations among key variables are shown in Table S1.

Table S1

*Correlation Matrix of Key Variables*

|  | Suffering | Deserve | Need | Moral | GBJW | Political |
| --- | --- | --- | --- | --- | --- | --- |
| Suffering | - |  |  |  |  |  |
| Deserve | .46*** | - |  |  |  |  |
| Need | .83*** | .56*** | - |  |  |  |
| Moral | .14** | .49*** | .25*** | - |  |  |
| Belief in a Just World | -.06 | -.08 | .02 | .11* | - |  |
| Political Ideology | -.03 | -.16** | .00 | -.10 | .46*** | - |
| Reward Likelihood | -.06 | .22*** | .01 | .23*** | .17** | .14** |

*Note.* * *p* < .05, ** *p* < .01, *** *p* < .001.

**Mediation analyses with sole mediators.** In our manuscript, we reported the results of mediation analyses where all three mediators were concurrently included. Here, we present the results of three mediation models, each with only one of the three mediators (moral character, deservingness, and need). Mediation analyses were conducted using R package *lavaan* version 0.6-3 (Rosseel, 2012) - standard errors were estimated with 5000 bootstrap draws. As seen in Figure S1b, the indirect effect of suffering on the perceived likelihood of winning through deservingness was significant and in the positive direction. There was also a significant direct effect in the opposite (i.e., negative) direction. As shown in Figures S1a and S1c, there was no significant indirect effect through moral character and need.

| **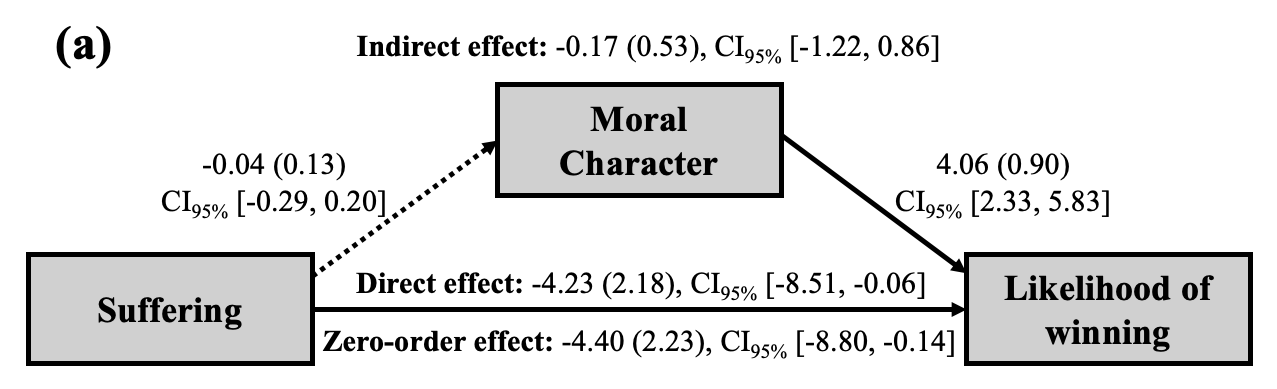** |
| --- |
| **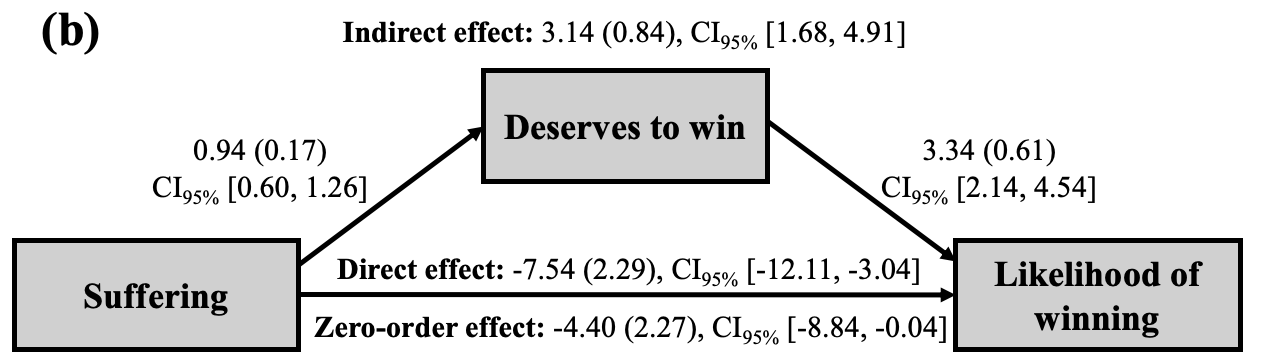** |
| **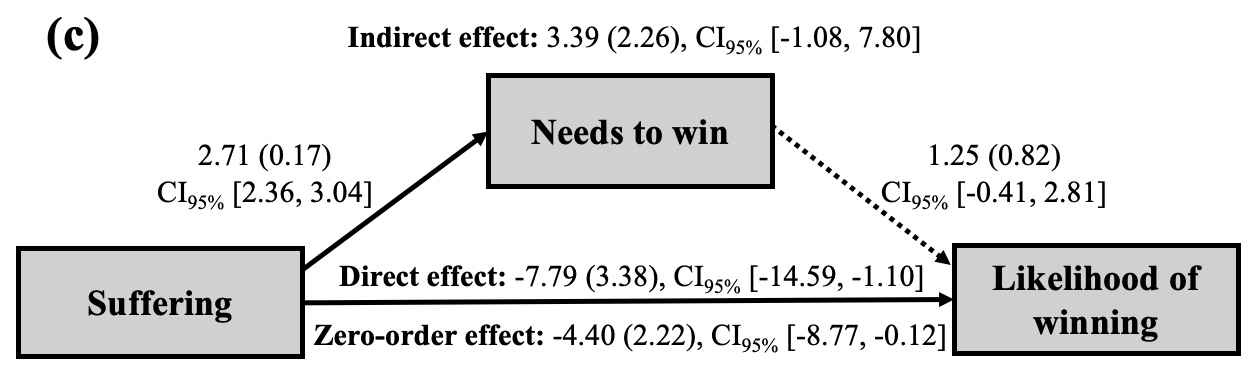** |

*Figure S1.* Results of mediation analyses with (a) moral character, (b) deservingness, and (c) need as mediators. Unstandardized coefficients shown with standard errors in parenthesis. Solid line denotes significant path while dashed line denotes non-significant path.

**Belief in a Just World.** The Global Belief in a Just World (GBJW) scale exhibited good internal consistency reliability (*α* = .95) for our sample.

***Effects of suffering manipulation on belief in a just world.*** Participants in both conditions did not significantly differ in their belief in a just world, suggesting that our manipulation did not significantly affect participants’ belief in a just world. An independent sample *t*-test showed that participants in the high and low suffering conditions did not differ in their belief in a just world (*M* = 3.14, *SD* = 1.32 vs. *M* = 3.28, *SD* = 1.31), *t*(365) = 1.05, *p* = .296, *d* = 0.11, CI_95%_ [-0.10, 0.31].

***Moderated mediation.*** To explore the potential role of belief in a just world in the illusory ‘suffering – reward’ association, we conducted a moderated mediation analyses (see Figure S2 for the conceptual model) using R package *lavaan* version 0.6-3 (Rosseel, 2012). The overall moderated mediation effect was significant, with an index of moderated mediation of -0.94 (*SE* = 0.49), CI_95%_ [-1.966, -0.002]. We found that the indirect effect through deservingness was stronger (i.e., more positive) for individuals lower (-1 *SD*) on the GBJW scale (4.34, *SE* = 1.12, CI_95%_ [2.35, 6.62]) as compared to individuals with a higher score (+1 *SD*) on the scale (1.86, *SE* = 0.95, CI_95%_ [0.15, 3.92]).

**
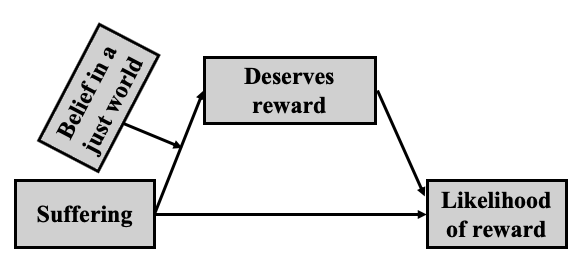
**

*Figure S2.* Conceptual model of the moderated mediation.

**Political Ideology.**

***Effect of suffering manipulation on political ideology.*** Participants in both conditions did not significantly differ in their political ideology, suggesting that our manipulation did not significantly affect participants’ political ideology. An independent sample *t*-test showed that participants in the high and low suffering conditions did not differ in their political orientation (*M* = 3.46, *SD* = 1.75 vs. *M* = 3.40, *SD* = 1.74), *t*(365) = 0.28, *p* = .779, *d* = 0.03, CI_95%_ [-0.23, 0.18].

***Moderated mediation.*** To explore the potential role of political ideology in the illusory ‘suffering – reward’ association, we conducted a similar moderated mediation analysis as we did for GBJW. We found a *trend* where the indirect effect through deservingness was stronger (i.e., more positive) for individuals on the left (-1 *SD*) of the political spectrum (4.21, *SE* = 1.04, CI_95%_ [2.31, 6.41]) as compared to individuals on the right (+1 *SD*) of the political spectrum (2.12, *SE* = 0.99, CI_95%_ [0.36, 4.26]). However, the overall moderated mediation effect was not significant, with an index of moderated mediation of -0.60 (*SE* = 0.35), CI_95%_ [-1.33, 0.07].

**Experiment 2**

**Method**

**Sample size computation.** Sensitivity power analysis conducted using G*Power (Faul et al., 2009) indicated that with a sample size of 450, we had 80% power to detect an effect size of *d* = 0.26 in a two-tailed independent sample *t*-test at an alpha level of .05. Sensitivity power analysis conducted an online application (Schoemann, Boulton, & Short, 2017) indicated that this sample size would also provide 80% power to detect an indirect effect in a one-mediator model where the correlation between (i) the predictor and mediator, and (ii) the mediator and outcome variable, are both *r* = .15. We deemed this level of power to be adequate. Our experiment contained four comprehension check questions and we *a priori* decided and preregistered that we would exclude participants who failed our comprehension check (i.e., answered less than three out of four questions correctly) from analyses. To account for exclusion, we adjusted our target sample size upwards by 20%, resulting in a target sample size of 540.

**Data collection.** The specifications for data collection were the same as that of Experiment 1. We did, however, encountered an unknown technical issue with TurkPrime and/or MTurk which prematurely terminated data collection, resulting in one less participant than intended (i.e., 539 participants in total).

**Results**

The correlations among key variables are shown in Table S2.

**Additional mediation analyses.** Here, we present the results of four mediation models, with suffering as the independent variable; deservingness, need, moral character, and unluckiness as sole mediators; and perceived likelihood of fortuitous reward as the outcome variable. Results are shown in Table S3. For both measures of reward likelihood, we found significant indirect effects in the positive direction through deservingness and need; along with a significant indirect effect in the negative direction through unluckiness. We also found a significant indirect effect in the negative direction through moral character (which was in the *opposite* direction of what the ‘virtuous suffering’ explanation would predict), though it was significant for the percentage measure but not for the 7-point measure.

**Belief in a Just World.** The GBJW scale showed good internal consistency reliability (*α* = .93) for our sample.

***Effects of suffering manipulation on belief in a just world.*** Participants in both conditions did not significantly differ in their belief in a just world, suggesting that our manipulation did not significantly affect participants’ belief in a just world. An independent sample *t*-test showed that participants in the high and low suffering conditions did not differ in their belief in a just world (*M* = 3.08, *SD* = 1.00 vs. *M* = 3.22, *SD* = 1.13), *t*(481) = 1.47, *p* = .142, *d* = 0.13, CI_95%_ [-0.04, 0.31].

***Moderated mediation.*** To explore the potential role of belief in a just world in the illusory ‘suffering – reward’ association, we conducted the same moderated mediation analysis we conducted for Experiment 1. The results of the moderated mediation are shown in Table S4. The results exhibited a similar pattern as that of Experiment 1. There was a significant moderated mediation effect where participants with lower scores on the GBJW scale exhibited a more positive indirect effect through deservingness.

Table S2

*Correlation Matrix of Key Variables*

|  | Suffering | Deserve | Unlucky | Need | Moral | Belief in a Just World | Reward likelihood  (percentage) |
| --- | --- | --- | --- | --- | --- | --- | --- |
| Suffering | - |  |  |  |  |  |  |
| Deserve | .47*** | - |  |  |  |  |  |
| Unlucky | .36*** | .27*** | - |  |  |  |  |
| Need | .64*** | .57*** | .28*** | - |  |  |  |
| Moral | .14** | .40*** | -.00 | .27*** | - |  |  |
| Belief in a Just World | .04 | .03 | -.04 | .03 | .11 | - |  |
| Reward likelihood (percentage) | .14** | .15** | -.10* | .14** | .13** | .14** | - |
| Reward likelihood  (7-point) | .12** | .13** | -.14** | .11* | .10* | .15** | .77*** |

*Note.* * *p* < .05, ** *p* < .01, *** *p* < .001.

Table S3

*Mediation Analyses with each Mediator Separately*

| Mediator | Likelihood  measure | Indirect effect | Direct effect | Zero-order effect |
| --- | --- | --- | --- | --- |
| Moral character | Percentage | -0.52* (0.31)  CI_95%_ [-1.24, -0.03] | 2.07 (1.89)  CI_95%_ [-1.58, 5.77] | 1.55 (1.89)  CI_95%_ [-2.10, 5.20] |
|  | 7-point | -0.03 (0.02)  CI_95%_ [-0.07, 0.00] | 0.03 (0.12)  CI_95%_ [-0.21, 0.25] | 0.00 (0.12)  CI_95%_ [-0.23, 0.23] |
|  |  |  |  |  |
| Deserves to receive surgery | Percentage | 1.35* (0.48)  CI_95%_ [0.54, 2.41] | 0.19 (1.97)  CI_95%_ [-3.56, 3.95] | 1.55 (1.95)  CI_95%_ [-2.18, 5.39] |
|  | 7-point | 0.07* (0.03)  CI_95%_ [0.02, 0.14] | -0.07 (0.12)  CI_95%_ [-0.30, 0.17] | 0.00 (0.12)  CI_95%_ [-0.22, 0.23] |
|  |  |  |  |  |
| Needs to receive surgery | Percentage | 1.90* (0.68)  CI_95%_ [0.70, 3.30] | -0.35 (2.04)  CI_95%_ [-4.35, 3.71] | 1.55 (1.94)  CI_95%_ [-2.25, 5.22] |
|  | 7-point | 0.10* (0.04)  CI_95%_ [0.02, 0.19] | -0.10 (0.13)  CI_95%_ [-0.34, 0.15] | 0.00 (0.12)  CI_95%_ [-0.22, 0.23] |
|  |  |  |  |  |
| Unlucky | Percentage | -1.15* (0.55)  CI_95%_ [-2.23, -0.14] | 2.70 (1.98)  CI_95%_ [-1.11, 6.47] | 1.55 (1.90)  CI_95%_ [-2.16, 5.13] |
|  | 7-point | -0.10* (0.04)  CI_95%_ [-0.17, -0.03] | 0.10 (0.12)  CI_95%_ [-0.13, 0.33] | 0.00 (0.12)  CI_95%_ [-0.23, 0.23] |

*Note.* Indirect effects marked with * indicate that the CI did not overlap with 0.

Table S4

*Results of Moderated Mediation with GBJW as Moderator*

| Dependent variable | Index of moderated mediation | Indirect effect through deservingness | |
| --- | --- | --- | --- |
|  |  | At -1 *SD* of GBJW | At +1 *SD* of GBJW |
| Reward likelihood (Percentage) | -0.62 (0.32)  CI_95%_ [-1.31, -0.05] | 2.03 (0.69)  CI_95%_ [0.83, 3.52] | 0.70 (0.44)  CI_95%_ [-0.02, 1.72] |
| Reward likelihood  (7-point) | -0.03 (0.02)  CI_95%_ [-0.088, -0.002] | 0.11 (0.05)  CI_95%_ [-0.03, 0.21] | 0.04 (0.03)  CI_95%_ [-0.00, 0.10] |

**Brief Discussion regarding Belief in a Just World**

Across Experiments 1 and 2, we found the same pattern of moderated mediation where the indirect effect through deservingness was more positive for individuals *lower* on the GBJW scale. We speculate that this set of findings might be a result of the GBJW scale predominantly reflecting the tendency to blame victims. For example, the scale consisted of items such as “I feel that people get what they deserve” and “I feel that people who meet with misfortunate have brought it on themselves”. Thus, participants who scored higher on GBJW might exhibit a greater tendency to blame the protagonist in our vignette, rating him as less deserving of future reward.

**Experiment 3**

**Method**

**Sample size computation.** Sensitivity power analyses indicated that with a sample size of 350 per condition (i.e., 1400 in total), we had (i) 91% power to detect an indirect effect assuming the pattern of associations shown in Figure S3, and (ii) 80% power to detect an effect size of *d* = 0.21 in an independent sample *t*-test at an alpha level of .05. We deemed this level of power to be adequate. Our experiment contained three comprehension check questions and we *a priori* decided and preregistered that we would exclude participants who failed our comprehension check (i.e., answered less than two out of three questions correctly) from analyses. To account for exclusion, we adjusted our target sample size upwards by 15%, resulting in a target sample size of 1610.

**Data collection.** Specifications for data collection were the same as that of Experiments 1 and 2 except that the Google RECAPTCHA was omitted in Experiment 3. While we had specified and paid for 1610 MTurk workers, we ended up with 9 additional participants (i.e., 1619 in total). According to TurkPrime’s FAQ page, this could be the result of participants not submitting the Human Intelligence Task (HIT) in time.

*
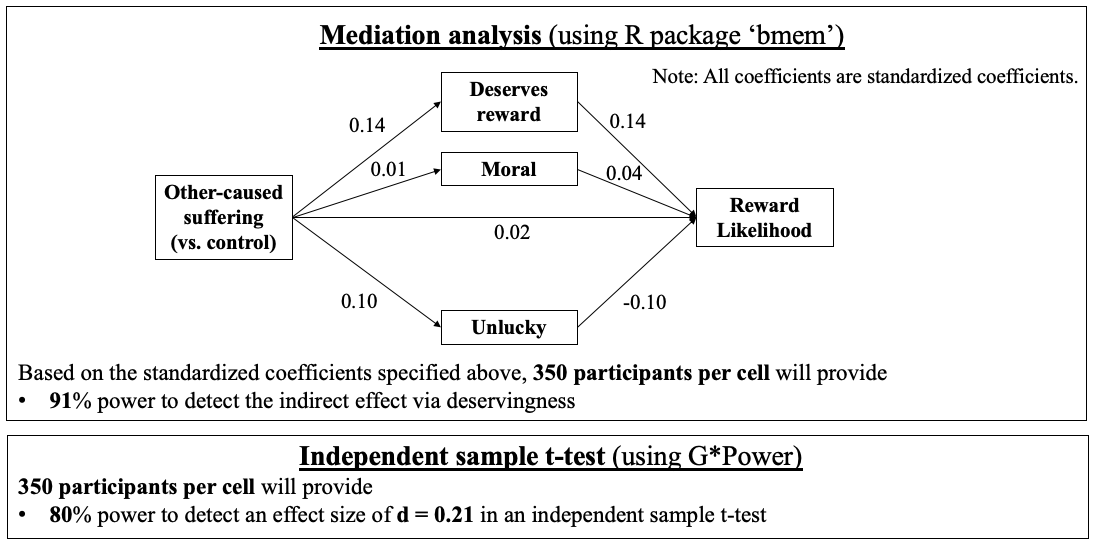
Figure S3.* Specifications for sensitivity power analyses for Experiment 3.

**Pretesting of vignettes.** To ensure the suitability of the vignette employed for Experiment 3, we first conducted a pretest (*n* = 100). We presented participants in the pretest with four potential vignettes (see Appendix A for these vignettes). Based on the results of the pretest, we selected the vignette we eventually used in Experiment 3. Further details about the pretest are available in the next section (pp. 15 - 19) of this document.

**Results**

The correlation matrix of the key variables in Experiment 3 is shown in Table S5.

Table S5

*Correlation Matrix of Key Variables*

|  | Likelihood | Deserve | Moral |
| --- | --- | --- | --- |
| Likelihood |  |  |  |
| Deserve | .30*** |  |  |
| Moral | .18*** | .49*** |  |
| Unlucky | -.00 | .05 | .04 |

*Note.* *** denotes *p* < .001

**Experiment 3 Pretest**

To assess the suitability of the vignette used in Experiment 3, we first conducted a pretest where we evaluated four vignettes. In this pretest, we focused solely on the other condition. This was because we were primarily concerned that participants might, contrary to our expectations, causally attribute what we conceived as ‘other-caused’ suffering to the victim or stochastic processes. To this end, we presented participants in this pretest with four potential vignettes (see Appendix A for these vignettes) and had them respond to several items for each vignette.

**Method**

Participants were 100 Amazon MTurk workers (*M*_age_ = 42.73, *SD*_age_ = 13.03; 55% males, 45% females). Specifications for data collection on TurkPrime was the same as that of Experiment 3. After providing consent, participants were presented with the first of four vignettes (see Appendix A). The protagonist in each vignette is experiencing suffering caused by other individual(s) and has the chance to experience a positive fortuitous outcome. After reading the vignette, participants rated how likely the protagonist will experience the positive fortuitous outcome on a 7-point scale (1 = *Very low chance*; 7 = *Very high chance*).

Next, participants rated (i) how much the protagonist deserves the positive outcome (1 = *Not at all*; 7 = *Very much*), (ii) how unlucky the protagonist is (1 = *Not at all unlucky*; 7 = *Extremely unlucky*). Next, we measured perceived moral character of the protagonist by having participants rate the protagonist on six traits (moral, principled, honest, trustworthy, fair, responsible) on a 7-point scale (1 = *Not at all*, 7 = *Very much*). The scale showed good internal consistency reliability (*α*s *≥* .94 for all four vignettes) in our sample. Then, to measure how participants’ causal attributions of the protagonist’s suffering, participants indicated the extent to which they agreed (1 *= Strongly Disagree*; 7 = *Strongly Agree*) that the suffering was (i) caused by other individual(s), (ii) caused by himself, and (iii) the result of coincidence.

The same procedures described above were repeated for the remaining three vignettes. The vignettes were presented in a randomized order. Finally, participants provided demographic information (i.e., age and gender).

**Results**

The descriptive statistics (i.e., means and standard deviation) are presented in Table S6. The correlation matrices for the four vignettes (i.e., Lost job, Burnt house, Blindness, Amputation) are presented in Tables S7 to S10 respectively.

Table S6

*Means of Key Variables in Experiment 3 Pretest*

|  | Vignette | | | |
| --- | --- | --- | --- | --- |
|  | Lost Job | Burnt House | Blindness | Amputation |
| Reward Likelihood | 3.94 (1.56) | 3.51 (1.59) | 4.45 (1.49) | 4.16 (1.34) |
| Mediators |  |  |  |  |
| Deserve | 5.37 (1.39) | 5.61 (1.51) | 4.86 (1.56) | 5.25 (1.12) |
| Unlucky | 4.65 (1.50) | 5.18 (1.59) | 4.44 (1.44) | 4.51 (1.59) |
| Moral character | 5.46 (1.07) | 5.52 (1.13) | 4.54 (1.17) | 5.28 (1.05) |
| Causal attribution |  |  |  |  |
| Other | 6.29 (1.32) | 6.48 (1.27) | 5.49 (1.49) | 5.60 (1.94) |
| Self | 2.09 (1.71) | 1.67 (1.46) | 3.60 (1.82) | 2.67 (1.88) |
| Coincidence | 2.47 (1.92) | 2.12 (1.78) | 3.05 (1.81) | 2.53 (1.87) |

*Note.* Standard deviations in parentheses.

Table S7

*Correlation Matrix of Key Variables in the ‘Lost Job’ Vignette*

|  | Likelihood | Deserve | Unlucky | Moral | Other | Self |
| --- | --- | --- | --- | --- | --- | --- |
| Likelihood |  |  |  |  |  |  |
| Deserve | .23 |  |  |  |  |  |
| Unlucky | .05 | .24 |  |  |  |  |
| Moral | .07 | .51 | .36 |  |  |  |
| Other | -.24 | .32 | .19 | .54 |  |  |
| Self | .41 | -.32 | -.03 | -.33 | -.66 |  |
| Coincidence | .36 | -.02 | .13 | -.12 | -.23 | .55 |

*Note.* Non-significant correlations coefficients are presented in gray.

Table S8

*Correlation Matrix of Key Variables in the ‘Burnt House’ Vignette*

|  | Likelihood | Deserve | Unlucky | Moral | Other | Self |
| --- | --- | --- | --- | --- | --- | --- |
| Likelihood |  |  |  |  |  |  |
| Deserve | .28 |  |  |  |  |  |
| Unlucky | .11 | .37 |  |  |  |  |
| Moral | .13 | .56 | .23 |  |  |  |
| Other | -.13 | .30 | .21 | .29 |  |  |
| Self | .32 | -.18 | -.05 | -.26 | -.48 |  |
| Coincidence | .19 | -.05 | -.09 | -.01 | -.38 | .56 |

*Note.* Non-significant correlations coefficients are presented in gray.

Table S9

*Correlation Matrix of Key Variables in the ‘Blindness’ Vignette*

|  | Likelihood | Deserve | Unlucky | Moral | Other | Self |
| --- | --- | --- | --- | --- | --- | --- |
| Likelihood |  |  |  |  |  |  |
| Deserve | .47 |  |  |  |  |  |
| Unlucky | .13 | .23 |  |  |  |  |
| Moral | .37 | .67 | .25 |  |  |  |
| Other | .14 | .37 | .29 | .44 |  |  |
| Self | .20 | -.15 | -.05 | -.23 | -.42 |  |
| Coincidence | .26 | -.07 | .28 | .08 | -.15 | .38 |

*Note.* Non-significant correlations coefficients are presented in gray.

Table S10

*Correlation Matrix of Key Variables in the ‘Amputation’ Vignette*

|  | Likelihood | Deserve | Unlucky | Moral | Other | Self |
| --- | --- | --- | --- | --- | --- | --- |
| Likelihood |  |  |  |  |  |  |
| Deserve | .39 |  |  |  |  |  |
| Unlucky | .02 | .00 |  |  |  |  |
| Moral | .29 | .56 | -.11 |  |  |  |
| Other | .16 | .42 | .08 | .35 |  |  |
| Self | .07 | -.25 | .12 | -.33 | -.73 |  |
| Coincidence | .29 | .04 | .24 | -.07 | -.27 | .47 |

*Note.* Non-significant correlations coefficients are presented in gray.

**Brief Discussion**

After inspecting the results, we concluded that the ‘Amputation’ vignette was most suitable for Experiment 3. This conclusion was arrived at through an elimination process.

We eliminated the ‘Lost job’ and ‘Burnt house’ vignettes for two key reasons. First, the correlation coefficients between deservingness and reward likelihood were relatively low for these two vignettes (*r*s = .23 and .28 respectively) and we postulated that a weaker (i.e. less positive) correlation coefficient could indicate a weaker indirect effect of suffering on reward through deservingness. Second, these two vignettes involved racism and a participant’s comment suggested that underlying racial tensions could introduce statistical noise in our data. For example, the indirect effect through deservingness might be higher among members of the racial minority and lower for individuals with negative attitude towards minorities.

We also eliminated the ‘Blindness’ vignette because there was a relatively greater tendency to causally attribute the suffering event to the self (*M* = 3.60). This posed a problem because we had expected that the indirect effect through deservingness would be weaker if the suffering was attributed to the self. This was also consistent with the finding that the deservingness rating was the lowest in this vignette (*M* = 4.86). This process of elimination left us with the ‘Amputation’ vignette. As we did not have any major concern with this vignette, we opted to use this vignette for Experiment 3.

Appendix A

Four vignettes used in Experiment 3 Pretest

| **Vignette #1: Lost Job**  Adam is a 32-year-old man who was, until several months ago, working as a customer service representative at a major telecommunication company. Like many of his colleagues, he was hired as a “contractor” rather than a full-fledged employee. Nonetheless, in the last four years, because his work performance was generally satisfactory, his contract was renewed every six months along with a small pay increment each time.  However, Adam got his contract terminated several months ago. This was because his team recently got a new direct supervisor who took an immediate dislike towards Adam because of Adam’s ethnicity. The supervisor did not publicly show his dislike but promptly terminated Adam’s contract citing several frivolous reasons.  After losing his job, Adam’s life swiftly fell into disarray. He started experiencing emotional problems, which hindered his ability to secure a new job, resulting in his savings becoming nearly depleted. He will soon be evicted from his small rental apartment and will end up on the streets.  Recently, Adam got to know about a new affordable housing initiative by the city’s housing office. Under this initiative, some affordable housing units are reserved for individuals below an income threshold. Successful applicants, determined by a random draw, will be able to rent apartments at a highly subsidized rate. Adam found out that he is eligible for this initiative and submitted an application. He believes that a successful application will help relief the financial pressure he is current facing. The initiative received a large number of applicants, with several hundred applicants for a few dozen units.  What do you think Adam’s chance of being allocated a housing unit in the random draw is? |
| --- |

| **Vignette #2: Burnt house**  Anthony is a 32-year-old single male working as a delivery driver for a major package delivery company. After saving up for years, he finally purchased his first home in the suburbs last year. He spent most of his savings on the down payment and had to take on a hefty mortgage in order to make the purchase.  A few evenings ago, his workplace organized an annual ‘employee appreciation party’ to thank Anthony and his colleagues for their hard work and effort. The party was held at a seaside resort and involve various games, performances and a delicious dinner.  There was also a raffle lottery where ten lucky employees will get to win a $5000 prize. Every employee was also given a raffle ticket during the party and Anthony’s raffle ticket number was #79. To build hype around the raffle lottery, it was decided that the random draw to determine the winners will only be held at a team meeting scheduled for the following week.  After the party, Anthony returned home to find a scene of chaos. His house was on fire with firefighters trying to douse the fire. He watched in despair as his house burned down along with most of his possessions. Subsequent investigation by the Police and Fire Departments revealed that the fire was set by a neighbor who hated Anthony for his ethnicity. This neighbor had since been arrested and charged with arson.  Following the fire, Anthony became homeless and had to stay in a homeless shelter in the city. The conditions in the homeless shelter is rather unfavorable. The living area is very crowded, and the beds are infested with bedbugs. He had tried to rent a room in a shared apartment, but he realized that he did not have enough money to pay for first month’s rent and the security deposit.  He then remembers that the raffle lottery draw will be conducted soon. As the $5000 prize would really help him get back on his feet, he hopes that he will be one of the winners of the random draw.  What do you think Anthony’s chance of being one of the winners is? |
| --- |

| **Vignette #3: Blindness**  Nick is a 22-year-old male. After he graduated from high school at age 18, he attended the local community college, majoring in business administration. However, he dropped out a year later after becoming disinterested in school. At that time, he got to know about the opportunities to earn extra cash by volunteering as a human test subject for clinical trials. These trials usually test drugs’ side effects and how it is processed in the human body. Such trials typically pay about $250 per day.  Nick had participated in four of such trials. The first three trials went without a hitch, but Nick experienced severe complications during the fourth trial. While he received prompt medical treatment, he still ended up with a permanent loss of vision in his right eye. The partial loss of sight resulted in Nick having difficulties judging distance and having frequent falls.  Subsequent investigation by the Food and Drugs Administration (FDA) found that the pharmaceutical company had engaged in fraudulent practices when seeking approval for human trials. More specifically, the company had covered up adverse findings from animal tests they previously conducted.  After which, he started looking for full-time employment and eventually took up an entry-level kitchen job at a fast-food chain. His income is only slightly above minimum wage and he is barely able to get by.  Recently, Nick came across a local news article which mentions that a non-profit organization is teaming up with a university to conduct a large-scale research study on ‘universal basic income’ in the small city he is living in. Universal basic income is a form of social security that provides every citizen with enough money to live on without any strings attached. The study aims to evaluate the effects of universal basic income on its recipients. Participants in the study will receive $1000 per month for three years and will be surveyed and interviewed by the researchers periodically.  The research study aims to recruit 1000 participants from all walks of life for the study. Nick is eligible and had applied to participate in the study. He believes that the $1000 per month income will vastly improve his quality of life. The research team will soon select participants amongst all the applicants via a random draw.  What do you think Nick’s chance of being selected for the research study is? |
| --- |

| **Vignette #4: Amputation**  Alan is currently a 21-year-old junior at university. He is majoring in French and has great interest in French literature and culture.  Alan is also a motorcycle enthusiast who frequently brought his fancy motorcycle out for a spin around his college town whenever he felt stressed out from school work. However, that changed sometime last year when he crashed his motorcycle into a tree. He got seriously injured and doctors had to amputate his left leg below the knee in order to save his life. Police investigation revealed that the crash was the result of a love rival sabotaging the brakes of Alan’s motorcycle.  The amputation took a heavy emotional toll on Alan. He has been experiencing great grief and is constantly worried about how other people will view him. Nonetheless, he resumed his education several months later.  Recently, Alan received a notice from the university informing students about the possibility to go for a 6-month study abroad program in France. Alan is very excited about this as he has dreamt of visiting France since high school. He believes that the trip will also help him immerse himself in the culture he has been learning so much about in class. He applied for the program and eagerly awaits the outcome.  It turns out that there are several dozen eligible applicants but only 8 vacancies. Therefore, the university will conduct a random draw to allocate the vacancies.  What do you think Alan’s chance of being selected for the study abroad program is? |
| --- |

References

Faul, F., Erdfelder, E., Buchner, A., & Lang, A.-G. (2009). Statistical power analyses using G*Power 3.1: Tests for correlation and regression analyses. *Behavior Research Methods*, *41*, 1149–1160. http://doi.org/10.3758/BRM.41.4.1149

Rosseel, Y. (2012). lavaan: An R package for structural equation modeling. *Journal of Statistical Software*, *48*, 1–36. http://doi.org/10.18637/jss.v048.i02

Schoemann, A. M., Boulton, A. J., & Short, S. D. (2017). Determining power and sample size for simple and complex mediation models. *Social Psychological and Personality Science, 8,* 379-386. https://doi.org/10.1177/1948550617715068
